# *Urtica dioica* Agglutinin Prevents Rabies Virus Infection in a Muscle Explant Model

**DOI:** 10.3390/pharmaceutics15051353

**Published:** 2023-04-28

**Authors:** Xinyu Wang, Lisanne Terrie, Guanghui Wu, Els J. M. Van Damme, Lieven Thorrez, Anthony R. Fooks, Ashley C. Banyard, Dirk Jochmans, Johan Neyts

**Affiliations:** 1 Department of Microbiology, Immunology and Transplantation, Rega Institute, KU Leuven, 3000 Leuven, Belgium; 2 Global Virus Network (GVN), Baltimore, MD 21201, USA; 3 Tissue Engineering Lab, Department of Development and Regeneration, Campus Kulak, KU Leuven, 8500 Kortrijk, Belgium; 4 Animal and Plant Health Agency (APHA), Woodham Lane, Weybridge KT15 3NB, UK; 5 Department of Biotechnology, Faculty of Bioscience Engineering, Ghent University, 9000 Gent, Belgium

**Keywords:** antiviral, rabies virus, lectin, muscle explant

## Abstract

Infection with the rabies virus (RABV) results in a 100% lethal neurological disease once symptoms develop. Post-exposure prophylaxis (PEP) consists of a combination of vaccination and anti-rabies immunoglobulins (RIGs); it is 100% effective if administered early after exposure. Because of its limited availability, alternatives for RIGs are needed. To that end, we evaluated a panel of 33 different lectins for their effect on RABV infection in cell culture. Several lectins, with either mannose or GlcNAc specificity, elicited anti-RABV activity, of which the GlcNAc-specific *Urtica dioica* agglutinin (UDA) was selected for further studies. UDA was found to prevent the entry of the virus into the host cell. To further assess the potential of UDA, a physiologically relevant RABV infection muscle explant model was developed. Strips of dissected swine skeletal muscle that were kept in a culture medium could be productively infected with the RABV. When the infection of the muscle strips was carried out in the presence of UDA, RABV replication was completely prevented. Thus, we developed a physiologically relevant RABV muscle infection model. UDA (i) may serve as a reference for further studies and (ii) holds promise as a cheap and simple-to-produce alternative for RIGs in PEP.

## 1. Introduction

Rabies virus (RABV), a member of the Lyssavirus genus, is a neurotropic virus that causes acute and fatal encephalitis in humans and animals. The incubation period in most patients is 20–90 days [1], and, upon the onset of symptoms, the disease is nearly 100% fatal. Post-exposure prophylaxis (PEP), in the form of vaccination and injection of rabies immunoglobulins (RIGs) in and around the wound shortly after exposure, can prevent infection [2]. However, RIGs are scarce in endemic regions [3,4] due to their short shelf-life, the need for a cold chain, and their high cost of manufacturing. It is estimated that less than 2% of severely exposed patients (category III) receive RIGs worldwide [5,6].

Humans are most commonly exposed to rabies when bitten by rabid animals with the infectious virus in their saliva. If the dermal barrier is breached, the virus infects muscle cells and gains access to neuronal cells through the neuromuscular junctions (NMJ). Once within the neuronal cells, the virus migrates from the peripheral nerves to the central nervous system (CNS), resulting in replication in the brain and the development of rabies. The furious form of the disease is characterized by an altered mental status, hydrophobia, aerophobia, or inspiratory spasms; whereas the paralytic form of the disease is characterized by flaccid muscle weakness and paralysis [7]. To enable cell entry, the nicotinic acetylcholine receptor (nAChR) serves as one of the key receptor molecules for rabies virus binding and entry [8,9,10]. However, the nAChR tends to be concentrated at the postsynaptic muscle membrane in the NMJ, which may drive the accumulation of rabies virions near the NMJ, enhancing the likelihood of the virus entering the peripheral neuron or motor neuron [11,12]. To this end, it is important to inhibit RABV accumulation or replication in muscle cells and prevent infection of the nervous system. This mechanism of action is proposed for RIGs, which are injected into the tissue near the exposure wound.

Lectins are a group of carbohydrate-binding proteins from plants, fungi, animals, or bacteria that can also be produced by recombinant DNA techniques [13]. Since the initial discovery of lectins more than 100 years ago, they became a focus of attention in many biological processes, including glycoprotein recognition, cell–cell communication, interaction with infectious agents, the recruitment of leukocytes to inflammatory sites, and tumor metastasis [13,14]. Several lectins, mostly with mannose and N-acetylglucosamine specificity, elicit antiviral activity against HIV through binding to gp120 [15,16] and the inhibition of viral entry [17]. HHA, a mannose-specific lectin from *Hippeastrum hybrid*, has been reported to inhibit SARS-CoV at the stage of viral attachment and fusion [18]. UDA, an N-acetylglucosamine-specific lectin from the rhizome of *Urtica dioica* (stinging nettle), is one of the smallest reported lectins, with a molecular weight of 8.5 kDa. Its two hevein domains are joined together by a short linker of four amino acids, and each of these domains includes a saccharide-binding site [19]. UDA has been reported to inhibit SARS-CoV-2 infection of cells by binding to the spike protein [20]. It also inhibits many enveloped viruses, including influenza A/B [21], dengue virus, and HIV-1/2 [19]. 

In this study, we identified several lectins that inhibit RABV infection in vitro. In order to evaluate their potential as an alternative to RIGs in a more physiologically relevant infection model, we established an RABV infection ex-vivo model using swine muscle strips. Our results demonstrate that UDA, a specific N-acetylglucosamine lectin derived from stinging nettle (*Urtica dioica*), efficiently inhibited RABV infection in this model. This study presents the first report on a physiologically relevant RABV muscle infection model and highlights the significance of our findings in the ongoing search for alternative solutions to RIG.

## 2. Materials and Methods

### 2.1. Cells, Virus, and Lectins

Rabies virus SAD-B19-mCherry was obtained from the laboratory of Professor Anthony Fooks (Animal & Plant Health Agency, Surrey, UK). The mCherry sequence is inserted before the first gene within the genome of the SAD B19 strain. Baby hamster kidney fibroblasts (BHK-21) cells (ATCC CCL-10™) were propagated in Dulbecco’s Modified Eagle Medium (DMEM) (Catalog number 41965039, ThermoFisher Scientific, Paisley, UK) with 10% fetal bovine serum (FBS) (Hyclone) and penicillin–streptomycin (P/S) (100 U/mL, ThermoFisher Scientific). When the cells were infected with RABV, 2% DMEM was used. Lectins were obtained from Professor Els Van Damme (Ghent University, Ghent, Belgium), then dissolved in PBS at 1 mg/mL, and stored at −20 °C. The (N-acetyl-D-glucosamine)n-specific lectins were extracted and purified from the rhizomes of the stinging nettle (*Urtica dioica*) by employing several rounds of affinity and/or ion-exchange chromatography. Purification was carried out in accordance with established protocols as described by Van Damme et al. [22]. The purity of the extracted lectins was confirmed to be over 99% by SDS-PAGE, indicating that the proteins were essentially pure.

### 2.2. Antiviral Assay

Compounds were added to cells in a 2-fold serial dilution from 100 μg/mL to 0.8 μg/mL, followed by virus at a multiplicity of infection (MOI) of 0.01 TCID_50_/cell. The antiviral activity was measured by the quantification of the mCherry fluorescence on day 5 post-infection (SPARK, Tecan, Mechelen, Belgium). The cell viability was determined by an MTS assay as described previously [23]. The half-maximal effective concentration (EC_50_) of the antiviral effect and the half-maximal cytotoxic concentration (CC_50_) were derived from the corresponding dose–response curves.

### 2.3. Time of Drug Addition Assay

**Figure 1:**
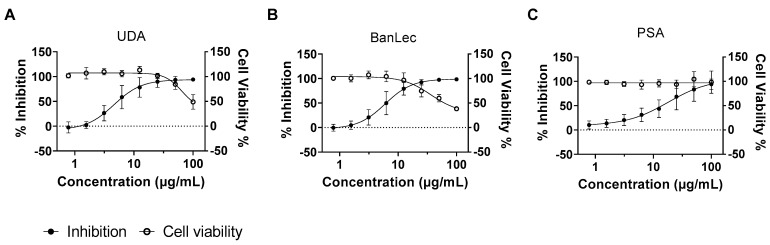
Anti-RABV activity of UDA, BanLec, and PSA in BHK-21J cells. Serial dilutions of UDA (**A**), BanLec (**B**), and PSA (**C**) were added together with mCherry-RABV (MOI = 0.01) to BHK cells. On day 5 p.i., the antiviral activity was determined by the quantification of the virus-induced mCherry fluorescence. In a parallel experiment, the effects of the lectins on cell viability were determined using a viability staining (MTS). The averages and standard deviations of 3 independent experiments are presented. Fitting the dose–response curves indicates an EC_50_ of UDA of 4.6 μg/mL and a CC_50_ of 65 μg/mL; an EC_50_ of BanLec of 6 μg/mL and a CC_50_ of 36 μg/mL; an EC_50_ of PSA of 18 μg/mL and a CC_50_ of more than 100 μg/mL.

**Figure 2:**
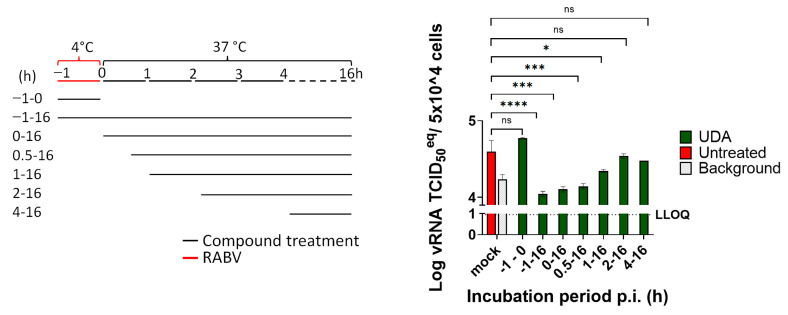
UDA blocks RABV entry. Time-of-drug-addition assay. BHK cells were incubated with RABV with or without UDA at 4 °C for 1 h (−1–0 h, indicated in red). After 1 h, unattached virus was washed away, and UDA was added to the cultures at different time points (0, 0.5, 1, 2, 4 h.p.i.). At 16 h.p.i., intracellular viral RNA was quantified by RT-qPCR. Infected and untreated samples collected at 1 h.p.i. were considered the virus background. Each condition was tested in 3 independent assays, and averages and STDEV are indicated. Tukey’s multiple comparisons test was used to calculate the statistical significance. (ns, not significant; * *p* < 0.05; *** *p* < 0.001, **** *p* < 0.0001).

UDA was utilized at a final concentration of 25 µg/mL, as it showed effective inhibition of the virus infection and no cytotoxicity, as demonstrated by the dose–response curve in Figure 1. To determine if UDA had an impact on the viral adsorption and attachment, cells were incubated with RABV (MOI = 1) at 4 °C for 1 h (−1–0 h), with or without UDA. After the incubation, to investigate if UDA inhibited viral entry, the unattached virus was washed away 3 times with PBS. Subsequently, the cells were incubated at 37 °C, and UDA was added at different time points (0, 0.5, 1, 2, and 4 h.p.i., Figure 2). The infected cells without compound treatment were defined as untreated controls, and the untreated samples collected at 1 hour post infection (h.p.i.) were considered the virus background. At 16 h.p.i., the viral RNA was extracted from the cells (E.Z.N.A. Total RNA Kit I R6834-01, Omega BIO-TEK) and quantified by RT-qPCR analysis.

### 2.4. Pre-Incubation of RABV to Lectins Prior to Infection

RABV (MOI = 0.4) was incubated with UDA (25 µg/mL) at 37 °C for 2 h. Then, the mixture of the virus and compound was diluted, added to BHK cells, and incubated at 37 °C (the virus final MOI was 0.1, and the UDA final concentration was 1 µg/mL). After 3 days, the viral mCherry fluorescence was quantified (SPARK^®^, Tecan, Belgium). The result was defined as “Pre-Incubation Virus”.

### 2.5. Pre-Incubation of BHK to Lectins Prior to Infection

The monolayer of BHK cells was pre-incubated with 25 μg/mL UDA at 37 °C for 2 h. Then, the compound was diluted by adding the virus suspension and then incubated at 37 °C (the virus final MOI was 0.1, and the UDA final concentration was 1 µg/mL). After 3 days, the viral mCherry fluorescence was quantified as above. This result was defined as “Pre-Incubation Cells”.

### 2.6. Swine Skeletal Muscle Explant Culture and Antiviral Assay

The biceps femoris muscle was dissected from freshly euthanized pigs (3 months, female), obtained as residual tissue from ORSI Academy (Melle, Belgium). The muscle biopsy was dissected parallel to the muscle fibers in 2 cm long and 2 mm thick strips using sterile scalpels. The tissue strips were maintained under tension using minutia pins (Entosphinx, stainless steel pins, diameter 0.25 mm) on sterile Sylgard-coated wells of a 6-well plate. During maintenance, the tissue strips were cultured (37 °C, 5% CO_2_) and submerged in DMEM with 10% FBS and P/S (100 U/mL) and in DMEM with 2% FBS and P/S during the antiviral experiments. For antiviral testing, the muscle explants were incubated with 25 µg/mL UDA (based on the results of the cell culture) at 37 °C for 2 h. Then they were inoculated with 2 × 10^4^ TCID_50_ RABV, while the UDA concentration was kept constant at 25 µg/mL. After 4 h of incubation, the muscle explants were washed 5 times with PBS and maintained with 25 μg/mL UDA for 6 days. For the untreated control, the muscle strips were inoculated with the same amount of virus but without the UDA treatment. A sample of 300 μL of the culture SN was collected every day for the untreated control for RT-qPCR analysis and on day 6 for the UDA-treated sample for the determination of the infectious virus. 

The tissue was obtained from a pig that was euthanized for robot surgery training, which was approved by the Ethical Committee of the ORSI Academy (EC2019/79). The laboratory number of the ORSI Academy is LA1400100. The use of residual tissue for research is also documented in the ethical approval.

### 2.7. Muscle Culture Supernatant Titration

The titer of the infectious virus, present in the swine muscle explant supernatant at day 6, was determined by 10-fold serial dilution on confluent BHK cells. On day 7, the virus-expressed fluorescence was quantified using microscopy, and the TCID_50_ was calculated by the Spearman–Kärber method.

### 2.8. Muscle Explant Viability Assay

The muscle strips were incubated with UDA at 25 µg/mL for 7 days. On each day, a resazurin-based toxicity assay was conducted: The medium of the muscle strips was removed and replaced with 1 mL of resazurin (PrestoBlue™ HS cell viability reagent P50200, ThermoFisher) working solution (10% PrestoBlue regent in PBS), followed by incubation at 37 °C for 2 h. After the incubation, 200 µL of resazurin working solution was transferred from the muscle culture plate to a transparent 96-well plate. The fluorescence was measured at an excitation of 560 nm and an emission of 590 nm. In living cells, the cell-permeable, non-fluorescent resazurin was reduced to red highly fluorescent resorufin. As resazurin is continuously converted into resorufin, the fluorescence is a quantitative indicator of the cell viability and cytotoxicity.

### 2.9. Fluorescent Immunostaining and Microscopy of Muscle

On day 6 p.i., swine skeletal muscle explants were washed 5 times with PBS, fixed in 4% formaldehyde for 1 h, and stored at 4 °C in PBS until staining. Before imaging, they were permeabilized with ice-cold 80% acetone at room temperature for 1 h and washed 5 times in PBS. Next, the muscle explants were incubated for 1 h at 37 °C with FITC Anti-Rabies Monoclonal Globulin (800-092, Fujirebio, Ghent, Belgium) diluted 1:10 in PBS. After washing them with PBS 5 times, the muscle explants were incubated in 5 µg/mL Hoechst (H1399, ThermoFisher Scientific, Paisley, UK) for 15 min at room temperature. Imaging was performed using the Leica DMi8 S Platform with Leica Application Suite X software (Leica, Heidelberg, Germany). All pictures were obtained with a 10× objective and z position between 1820 µm and 1950 µm.

### 2.10. Real-Time RT-qPCR

The rabies virus N gene was amplified by real-time quantitative PCR using the iTaq™ Universal SYBR^®^ Green One-Step Kit (BIO-RAD). The primers were as follows: 5′-TGGGCACAGTTGTCACTGCTTA-3′ (forward) and 5′-CTCCTGCCCTGGCTCAA-3′ (reverse). The standard curve was generated from the RNA extraction of the 1/10 diluted virus stock (2.2 × 10^6^ TCID_50_/mL). A 20 μL qPCR reaction contained 4 μL of extracted sample RNA or standard, 10 μL of iTaq Universal SYBR^®^ Green reaction mix, 0.25 μL of reverse transcriptase, and 600 nM of each forward and reverse primer. qPCR was performed in a Roche LightCycler 96 with the following procedure: 10 min at 50 °C for reverse transcription, 1 min at 95 °C for polymerase activation and DNA denaturation, 40 cycles of 95 °C 15 s and 62 °C 30 s for PCR amplification. Viral copies were calculated based on a standard curve using control material. 

### 2.11. Statistics

Statistical analysis in this study was performed using GraphPad Prism 9. Tukey’s multiple comparisons test and the Kruskal–Wallis test with Dunn’s multiple comparisons test were used to calculate the statistical significance. A *p*-value less than 0.05 was considered statistically significant. *p*-values associated with each graph are indicated as: *, *p*-value < 0.05; **, *p*-value < 0.01; ***, *p*-value < 0.001; ****, *p*-value < 0.0001. 

## 3. Results

### 3.1. Several Lectins Inhibit RABV in Cell Culture

The effects of 33 lectins on the RABV infection of BHK-21 cells were assessed using a reporter virus (SAD-B19-mCherry). Several lectins that elicited antiviral activity were identified (Table 1). 

The GlcNAc-specific agglutinin (*Urtica dioica* agglutinin (UDA)), the mannose-specific BanLec from *Musa acuminata* (banana), and the PSA from *Pisum sativum* (pea) were the most potent and selective. These activities were confirmed in a follow-up experiment with the same method as the screening (EC_50_s UDA: 4.6 µg/mL; BanLec: 6.0 µg/mL; PSA: 18 µg/mL) (CC_50_s UDA: 65 µg/mL; BanLec: 36 µg/mL; PSA: >100 µg/mL) (Figure 1). 

UDA (which we had available in larger quantities), was selected for further study. To explore at which step of the virus replication cycle UDA exerts its activity, the lectin was added to the infected cultures at various times pre- and post-infection. When UDA (25 µg/mL) was only present during the binding process at 4 °C (from time −1 h–0 h.p.i.), no antiviral effect was measured. When the lectin was present at 37 °C (a temperature that enables virus entry) between 0 and 1 h.p.i., the lectin prevented the RABV infection of cells. When the addition of UDA to the cultures was delayed for one or two hours p.i., a gradual loss in the antiviral activity was noted (Figure 2). 

**Figure 3:**
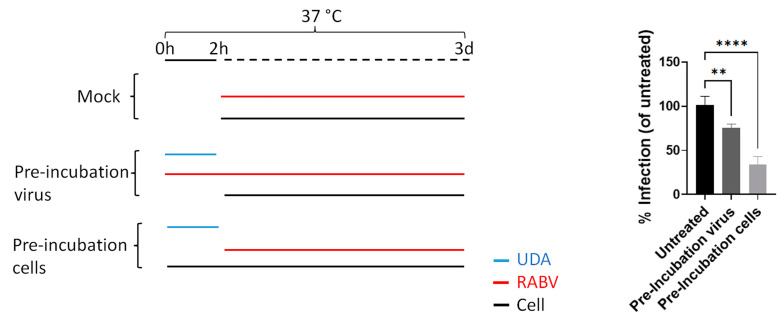
UDA inhibits RABV infection by mainly interacting with the cell. UDA at a final concentration of 25 µg/mL was pre-incubated with RABV or cells at 37 °C for 2 h. Next, UDA was diluted to a non-inhibitory concentration (1 µg/mL), and cells were further incubated with virus (MOI 0.1) for 3 days at 37 °C. Virus infection (%) was determined relative to the untreated condition. Each condition was tested in at least 3 independent experiments, and averages and STDEV were calculated. Tukey’s multiple comparisons test was used to calculate the statistical significance. (** *p* < 0.01, **** *p* < 0.0001).

Together, the results indicate that UDA blocks RABV replication at the entry but not the binding step. To investigate whether UDA interacts with the virus or the cells, both the virus and cells were pre-incubated with 25 µg/mL UDA at 37 °C for 2 h, after which the mixture was diluted to a non-inhibitory concentration (1 µg/mL) and mixed with the cells and virus, respectively. The RABV infection was quantified by measuring the viral fluorescence signal at 3 days p.i. Pre-incubation of the cells with UDA was more efficient in blocking the viral signal (*p* < 0.0001) than when the virus was preincubated with UDA, indicating that the predominant mechanism of action may be the results of an interaction of the lectin with the host cell (Figure 3).

### 3.2. RABV Replicates in Swine Skeletal Muscle Explants

**Figure 4:**
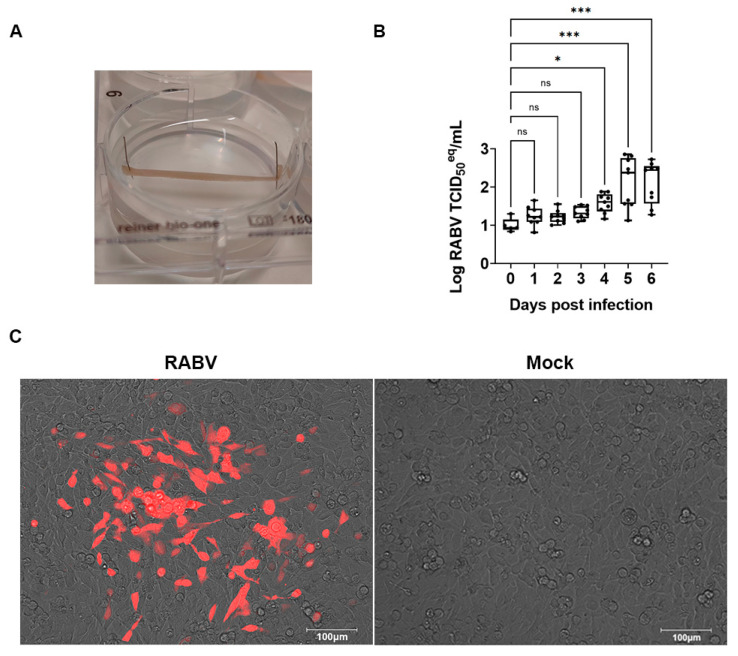
RABV replicates in swine skeletal muscle explants. (**A**) Picture of a swine muscle explant culture. The biceps femoris muscle was dissected from freshly euthanized pigs (3 months, female) and maintained under tension using pins. (**B**) Upon infection with RABV (SAD-B19-mCherry), culture supernatant was analyzed each day for levels of vRNA by RT-qPCR. Nine independent cultures were used; median and quartiles are indicated. Kruskal–Wallis test with Dunn’s multiple comparisons test was used to calculate the statistical significance. (ns, not significant; *, *p*-value < 0.05; ***, *p*-value < 0.001) (**C**) On day 6 p.i., the supernatants of the infected (RABV) and non-infected (Mock) muscle cultures were transferred to BHK cells, and after 7 days of incubation, the fluorescence of the cells was visualized by microscopy (the picture is a representative culture of 3 independent experiments). Scale bar: 100 µm.

Biceps femoris muscle samples (2 cm long, 2 mm thick) were dissected from freshly euthanized 3-month-old female pigs and maintained ex vivo under tension using pins (Figure 4A).

In the first step, we explored whether RABV can replicate in this muscle tissue. The explant cultures were inoculated with 2 × 10^4^ TCID_50_ of virus for 4 h. Next, the viral inoculum was removed, and the muscle strips were washed 5 times with PBS. The culture supernatant was collected every day for 6 consecutive days to determine the viral RNA load. On day 5–6 post-infection, the viral RNA in the culture supernatant was >10-fold higher as compared to day 0 (Figure 4B). To confirm that infectious virus was produced, the culture medium obtained from the muscle cultures on day 6 was used to inoculate BHK cells. The mCherry red fluorescence expressed by the virus was observed in BHK cells infected with this day 6 inoculum (Figure 4C), demonstrating that swine skeletal muscles can be productively infected with RABV.

### 3.3. UDA Inhibits RABV Infection in Swine Skeletal Muscle

**Figure 5:**
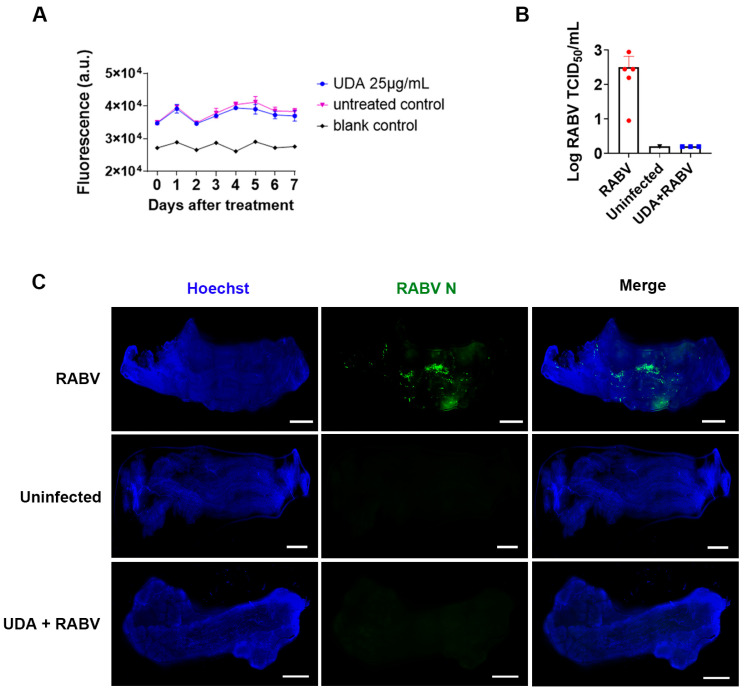
UDA inhibited RABV replication in swine skeletal muscle explants. (**A**) Viability (using resazurin) of muscle explants that were incubated for 7 days with UDA (25 µg/mL) at 37 °C. The resazurin-based viability assay was conducted daily. Blank control: cultures without muscle strips. Three independent cultures were used; averages and STDEV are indicated. (**B**) Muscle explants were infected with RABV in the presence or absence of UDA (25 μg/mL). At 6 d.p.i. the culture supernatant was collected to determine infectious viral titers. (**C**) On day 6 p.i., the muscle strips were fixed, and RABV replication was visualized by staining for the viral N-protein (green); cell nuclei were stained with Hoechst (blue). Results of a representative example of 3 independent experiments are shown. Scale bar: 1 mm.

To investigate whether UDA can prevent the RABV infection of muscle tissue, the muscle strips were pre-incubated with UDA (25 μg/mL) for 2 h, followed by virus inoculation in the presence or absence of UDA. After 4 h, the virus inoculum was removed, and new medium, with or without UDA (25 μg/mL), was added. On day 6 post-infection, the culture supernatant was collected for titration on BHK cells, and the muscle tissues were fixed for immunofluorescence staining. The UDA treatment resulted in a significant decrease in virus production in the medium (Figure 5B) and in antigen expression in the cells (Figure 5C). At a concentration of 25 µg/mL, UDA had no adverse effect on the viability of the muscle strips during the 7-day exposure period (Figure 5A).

## 4. Discussion

Rabies immunoglobulins (RIG) are used, together with vaccination, in RABV post-exposure prophylaxis (PEP) after high-risk exposure. RIGs provide passive protection by neutralizing the virus in the wound and surrounding tissues. Due to the high cost and supply shortages of RIGs, the WHO has recommended, since the 1990s, the development of alternatives [24]. Some monoclonals have been licensed for the Indian (Rabishield and Twinrab) [25] and Chinese (Ormutivimab) markets, but they obviously suffer from the same shortcomings [5]. 

In an attempt to develop an alternative for RIGs, we explored whether a series of lectins (with various specificities) that are easy and cheap to produce, and which do not require a cold chain, may prevent the entry of RABV in cells. We identified that the GlcNAc-specific agglutinin UDA from *Urtica dioica* (stinging nettle), the mannose-specific lectins BanLec from *Musa acuminata* (banana), and PSA from *Pisum sativum* (pea) were the most potent and selective inhibitors of virus infection within this series. UDA (which was readily available to us) was selected for further studies. Others have reported that the mannose/glucose-specific lectin Concanavalin A (con A) has prevented RABV entry in cells [26], but we observed that the antiviral and cytotoxic effects of con A were nearly overlapping. Time-of-drug-addition studies and an experiment in which either the virus or the cells were preincubated with UDA indicate that this lectin prevented entry (not binding) of the virus in the cells and did so predominantly by interacting with the host cell. Lectins are characterized by their reversible binding to a specific mono- or oligosaccharide [27]. Several lectins have been reported to exert anti-bacterial and antiviral activities [28], and some lectins have been shown to elicit antiviral activity by binding with viral glycans of enveloped viruses [15,16,18,20,29], such as HIV and SARS-CoV2. Here, in contrast, we found that the mechanism by which UDA inhibits RABV infection stems primarily from an interaction with the host cells. Seganti et al. [30] reported that treating *Aedes pseudoscutellaris* cells with wheat germ agglutinin (WGA) and Ricinus communis agglutinin (RCA-I), which are lectins with specificity for N-acetylglucosamine and galactose, respectively, resulted in the significant inhibition of rabies virus attachment. This indicates that the involvement of N-acetylglucosamine and galactose is crucial in the early stages of RABV infection. In our study, UDA may have also interacted with the cell in a way that prevented RABV entry but not binding. 

Since RABV typically replicates in muscle tissue upon a bite from a rabid animal and before the virus enters the nervous system, we aimed to develop a physiologically relevant RABV infection model in muscle explants. We isolated intact swine skeletal muscles and placed them under tension in a culture medium. The muscles remained metabolically active for at least 6 days. When these muscle strips were infected with RABV, viral antigens were detectable in the tissue, and infectious virus particles were released in the culture medium. This demonstrates that RABV infects and productively replicates in swine muscle explants. We then used this physiologically relevant model to demonstrate that UDA can completely block RABV infection and subsequent replication in these muscle explants. While it would be difficult to simulate a wound or RIG injection in muscle strips, our results demonstrate that UDA effectively blocked the viral infection. Based on this, it can be presumed that if UDA is deeply injected into the muscles, it will also effectively prevent virus spread. Thus, the activity of UDA against RABV, as observed in BHK cells, also translates to muscle tissue, demonstrating that this, and possibly other lectins, may be further studied as alternatives for RIGs. Next, the efficacy of UDA, or ideally, a lectin with more activity against RABV, or a combination of different lectins, should be assessed in rodent models of RABV infection.

The immunogenicity of lectins is highly variable and depends on the source of the lectin. The interaction of plant lectins with glycan moieties present on the surface of immune cells initiates immune-modulatory activities for several lectins. UDA has been described to have immuno-modulatory activity [31,32], and it can be considered a harmless herbal compound without toxicity to the host macrophages [33]. In the context of post-exposure prophylaxis for rabies, it can be assumed that a lectin (not necessarily UDA, which was used here for proof-of-concept) is expected to be administered only once in a patient’s lifetime to prevent a fatal rabies infection. Therefore, the development of a neutralizing antibody response against the lectin is not expected to cause any issues.

In conclusion, we have developed a novel, physiologically relevant RABV infection model using swine skeletal muscle explants. Our results demonstrate the potential of plant lectins, such as UDA, in preventing RABV infection in vitro and in an ex vivo muscle model. UDA, as well as other lectins, may have the potential to serve as a valuable reference for future studies in the development of alternative options for post-exposure prophylaxis against rabies.

## Figures and Tables

**Table 1:** Antiviral activity of lectins against RABV.

Lectin	Species	^1^EC_50_ (μg/mL)	CC_50_ (μg/mL)	SI
Gal/GalNAc-specific agglutinins				
PHA-L4	*Phaseolus vulgaris*	47 ± 13	>100	>2.1
RPA	*Robinia pseudoacacia*	5.4 ± 2.5	11 ± 1.4	2.1
RSA	*Rhizoctonia solani*	>100	>100	Na
SJA	*Styphnolobium japonicum*	32 ± 19	>100	>3.2
GalNAc-specific agglutinins				
BPA	*Bauhinia purpurea*	3.2 ± 1.0	9.0 ± 2.1	2.8
CAA	*Caragana arborescens*	95 ± 5	>100	>1.0
DBA	*Dolichos biflorus*	>100	>100	Na
SBA	*Soybean*	>100	>100	Na
WFA	*Wisteria floribunda*	>100	>100	Na
Gal-specific agglutinins				
Jacalin	*Jackfruit*	>100	>100	Na
Morniga G	*Morus nigra*	>100	24 ± 8.6	<0.2
PHA-E	*Phaseolus vulgaris*	47 ± 1.5	86 ± 18	1.8
PNA	*Arachis hypogaea*	>100	>100	Na
GlcNAc-specific agglutinins				
DSL	*Datura stramonium*	57 ± 2.2	69 ± 12.4	1.2
GS II	*Griffonia simplicifolia*	>100	>100	Na
Nictaba	*Nicotiana tabacum*	29.0 ± 3.9	>100	>3.4
UDA	*Urtica dioica*	8.1 ± 1.3	54 ± 6.8	6.7
UEA II	*Ulex europaeus*	>100	>100	Na
WGA	*Wheat germ*	21 ± 7.0	17 ± 6.3	0.8
Man/GalNAc-specific agglutinins				
AMA	*Arum maculatum*	7.1 ± 2.9	9.1 ± 2.2	1.3
TLC I	*Tulipa hybrid*	32 ± 5.7	>100	>3.2
Mannose-specific agglutinins				
APA	*Allium porrum* L.	36 ± 11	>100	>2.9
Banana lectin	*Musa acuminata*	6.2 ± 2.6	40.7 ± 11.5	6.5
Calsepa	*Calystegia sepium*	>100	>100	Na
Con A	*Canavalia ensiformis*	13 ± 3.7	22 ± 4	1.7
Conarv A	*Convolvulus arvensis*	90.7 ± 9.3	>100	>1.1
GNA	*Galanthus nivalis*	>100	>100	Na
HHA	*Hippeastrum hybrid*	29 ± 2.3	>100	>3.4
Morniga M	*Morus nigra*	>100	27.4 ± 14.1	<0.3
NPA	*Narcissus pseudonarcissus*	16 ± 5.4	42 ± 5.8	2.6
PSA	*Pisum sativum*	13 ± 7.5	>100	>7.7
Sialic acid-specific agglutinin				
ACA	*Amaranthus caudatus*	>100	>100	Na
MAA	*Maackia amurensis*	>100	>100	Na

Man: mannose; GlcNac: N-acetyl glucosamine; GalNAc: N-acetyl galactosamine; Gal: galactose; Neu5Ac: N-acetylneuraminic acid; SI: selectivity index; Na: not determined ^1^EC_50_ and CC_50_ are reported as the average and SD of 3 independent experiments.

## Data Availability

Not applicable.
